# First District Dental Society of New York

**Published:** 1904-05

**Authors:** B. C. Nash

**Affiliations:** Secretary


### Domestic Correspondence.

### FIRST DISTRICT DENTAL SOCIETY OF NEW YORK.

To the Editor:

Sir,—In the April number of the International, at page 320, appears a notice of election of officers of the First District Dental Society of New York, under my name as Secretary.

Will you kindly inform me who is responsible for the notice? I did not send it or authorize it, and it is incorrect, as it is in advance of the annual meeting, which will not be held until the 12th inst. It is quite likely that some one will criticise this premature action, and I want to be in a position to place the responsibility where it belongs.

B. C. Nash,
*Secretary.*

[We regret that it is not possible to comply with the reasonable wish of our correspondent. Copy of this character is not preserved. When these notices are received they are supposed to be authorized, and are published as sent.—Ed.]

